# Mast cell distribution and prevalence in the murine urinary bladder

**DOI:** 10.1186/s12894-024-01435-6

**Published:** 2024-03-05

**Authors:** Jessica Smith, Jonathan Kah Huat Tan, Christian Moro

**Affiliations:** 1 Clem Jones Centre for Regenerative Medicine, Bond University, Queensland, 4226 Australia; 2 Faculty of Health Sciences and Medicine, Bond University, Queensland, 4226 Australia

**Keywords:** Mast cell, Urinary bladder, Flow cytometry, Toluidine blue, CD117, FcɛRIα, CD45

## Abstract

**Background:**

Mast cells have been implicated in the pathology of various urinary bladder disorders. However, the distribution of mast cells throughout urinary bladder tissue remains uncertain despite mast cell prevalence being relatively well-defined. Using a mouse tissue model, this study aims to characterise the prevalence and distribution of mast cells throughout the urinary bladder.

**Methods:**

Bladder tissues were collected from six C57BL/6J female mice. Mast cell prevalence was quantified by flow cytometry, based on the expression of the following characteristic markers: CD45, CD117 and FcɛRIα. The toluidine blue stain assessed mast cell distribution, size, and proximity to vasculature. A repeated measures one-way ANOVA was used to evaluate the density of mast cells between the discrete layers of the urinary bladder, and an ordinary one-way ANOVA was used to assess potential differences between mast cell size across the urinary bladder wall.

**Results:**

It was determined that mast cells compose less than 4% of all live leukocytes in the urinary bladder. They were also found to be more prominent in the lamina propria and detrusor muscle layers, compared to the urothelium and adventitia. In addition, 20.89% of mast cells were located near vasculature, which may be an important factor in consideration of their function and potential to contribute to various bladder pathologies, such as cystitis or overactive bladder.

**Conclusion:**

These findings provide a baseline understanding of mast cell prevalence and distribution throughout the urinary bladder.

## Background

Mast cells are heterogenous, tissue-resident leukocytes present in most vascularised tissues around the body [1]. They sit as key mediators of both innate and adaptive immunity [2] and play important roles in various pathologies around the body, such as in allergies, inflammation [2, 3] and clonal mast cell disorders [4]. Mast cells undergo maturation outside the bone marrow, in peripheral tissues [5], under the influence of microenvironmental growth factors such as stem cell factor [6]. Most mast cell progenitors express the stem cell factor receptor *c-kit* (CD117) and the high-affinity immunoglobulin E receptor FcɛRIα, which maintain their expression throughout maturation [5]. These antigens, alongside the common leukocyte antigen (CD45), serve as markers for the identification and quantification of mast cells by flow cytometry [7, 8]. The use of antigens, including CD117, FcɛRIα and CD45 has enabled the identification and quantification of mast cells, allowing for a better understanding of their role in diseases.

In the urinary bladder, mast cells have been implicated in the pathogenesis of various disorders, such as overactive bladder [9, 10], bladder outlet obstruction [11, 12], urinary tract infections [13], bladder carcinoma [14] and various forms of cystitis, namely interstitial cystitis [10, 15, 16], radiation-induced cystitis [17] and bacterial cystitis [18]. In such pathologies, abnormalities have been reported in their activation state, prevalence and distribution. For example, in the healthy human urinary bladder, mast cells are reported to be distributed throughout the urothelium, lamina propria and detrusor muscle layers [15, 19]. Mast cells are noted to be in particularly low quantities in the urothelium and lamina propria [10] and are also reported to be in close proximity to vasculature [20]. In interstitial cystitis, several reports have indicated the involvement of mast cells in the pathology of this disease by demonstrating large numbers of activated and degranulated mast cells [19, 21–23]. In particular,  a comprehensive study conducted by Theoharides, Kempuraj [23] found that in the detrusor of non-ulcerative interstitial cystitis, mast cell prevalence increased 6- to 8-fold higher compared to control data, whereas in ulcerative interstitial cystitis mast cell infiltration was 2- to 3-fold higher compared to non-ulcerative interstitial cystitis [23]. A significant increase of mast cells was also observed in the mucosa and submucosa in non-ulcerative interstitial cystitis, however it was noted that mast cell infiltration in this layer occurs to a lesser extent than that of the detrusor muscle [23].

Despite the available literature on mast cell distribution in the human urinary bladder, current literature relating to mast cell distribution in murine models of disease remains sparse. Mice are commonly used to model immunological pathologies, such as cystitis [8, 24, 25], though comprehensive data relating to the prevalence and distribution of mast cells in the murine urinary bladder is lacking. It is possible that changes to mast cell prevalence and distribution may be a contributing factor to a variety of urinary bladder pathologies, warranting exploratory investigation into baseline data. As such, this study aims to assess the prevalence and distribution of mast cells across the urinary bladder wall.

## Methods

### Animals

Female C57BL/6JArc (C57BL/6J; CD45.2) mice were acquired from Animal Resource Centre (Perth, Western Australia, Australia) and housed at the Bond University animal holding facility.

### Ethics approval

Mice were housed and handled according to standard operation protocols approved by the University of Queensland Animal Ethics Committee. Euthanasia was performed by cervical dislocation. Animal ethics approval was granted by the University of Queensland Molecular Biosciences Animal Ethics Committee under a shared tissue agreement (BOND/ANRFA/162/20).

### Cell counting

Cell numbers were estimated by staining single-cell suspensions with trypan blue (12% saline) (Sigma-Aldrich Corporation; St. Louis, Missouri, U.S.A.) at a 1:2 dilution. Cells were transferred onto a haemocytometer (Electron Microscopy Sciences; Hatfield, Pennsylvania, U.S.A.). Live cells unstained by trypan blue were counted by phase-contrast microscopy using a Diavert Inverted microscope (Leica Microsystems GmbH; Wetzlar, Germany).

### Flow cytometry

Flow cytometry was used to identify and quantify mast cells from six urinary bladders. Cells were aliquoted into a 96-well U-bottom plate (TPP Techno Plastic Products AG; Trasadingen, Switzerland), centrifuged for five minutes at 4 °C for 200G, after which the supernatant was discarded. Cells were resuspended in 1.5mL of PBS and dispersed across a 96-well plate. Wells were labelled with 10µL antibody solution, prepared by diluting each antibody 1:100 in staining buffer. 10µL of antibody solution was added to each well and then incubated for 10 min on ice, protected from light. Stained cells were then washed with 150µL of staining buffer. Samples were then resuspended in 150µL of staining buffer and transferred into 5mL falcon round-bottom tubes (Becton, Dickinson and Company; Franklin Lakes, New Jersey, U.S.A) for flow cytometry analysis.

Propidium iodide (Sigma-Aldrich Corporation) was used to discriminate between live and dead cell populations. CD45, a pan-leukocyte marker that can detect almost all haematopoietic derived cells [26], was used to contextualise mast cell prevalence out of all leukocytes in the urinary bladder. Mast cell markers are described below (Table 1).

**Table 1:** **Experimental markers.** aF647 = Alexa Fluorochrome 647 dye; PB = Pacific Blue; PE = phycoerythrin. All markers were supplied by BioLegend

Antibody	Fluorochrome	Clone	Concentration
FcεRIα	PE	MAR-1	0.2 mg/mL
CD117	aF647	2B8	0.5 mg/mL
CD45.2	Pacific Blue	104	0.5 mg/mL

Flow cytometry analysis was performed using BD FACSAria™ Fusion III (Becton Dickinson). Single-colour controls were used to adjust compensation for spectral overlap, and cell population gating was based on a series of fluorescence minus-one control antibody mixtures. Flow cytometric data was analysed using FlowJo (FlowJo v10.8.1; FlowJo LLC; Ashland, Oregon, U.S.A.).

### Toluidine blue staining

Toluidine blue was used to identify mast cells in the urinary bladder. Tissue was harvested and prepared from six C57BL6/J mice. Tissue was fixed in 10% neutral buffered formalin (Sigma-Aldrich Corporation) prior to being snap-frozen in Tissue-Tek O.C.T. compound (Sakura Finetek USA Inc; Torrance, California, U.S.A). Three 10 μm tissue sections were taken per sample and placed onto Starfrost advanced adhesive slides (ProSciTech; Kirwan, Queensland, Australia). Sections were first rehydrated through sequential 5-minute immersions in 100%, 95%, and 70% ethanol before being placed in distilled water for 2 min. Slides were then immersed being submersed in toluidine blue for 3 min. Slides were then sequentially dehydrated in 70% ethanol, 95% ethanol (twice) and 100% ethanol (twice) for two minutes each. Tissue were imaged using a live cell microscope equipped with a brightfield camera (Nikon Eclipse Ti2-E; Nikon Corporation; Tokyo, Japan).

### Statistics

Mast cell density was reported out of all three sections per sample by mm^2^ of bladder tissue and proximity to vasculature was determined if the mast cell was situated less than 10 μm away from the nearest blood vessel.

A repeated measures one-way ANOVA was used to assess the density of mast cells between the discrete layers of the urinary bladder, and an ordinary one-way ANOVA was used to assess potential differences between mast cell size across the urinary bladder wall. All other data was presented as descriptive statistics, reported as mean±SEM. Statistics were performed using Prism 9 for macOS (v9.3.0, GraphPad Software; San Diego, California, U.S.A.).

## Results

### Mast cell prevalence in the urinary bladder

**Fig. 1:**
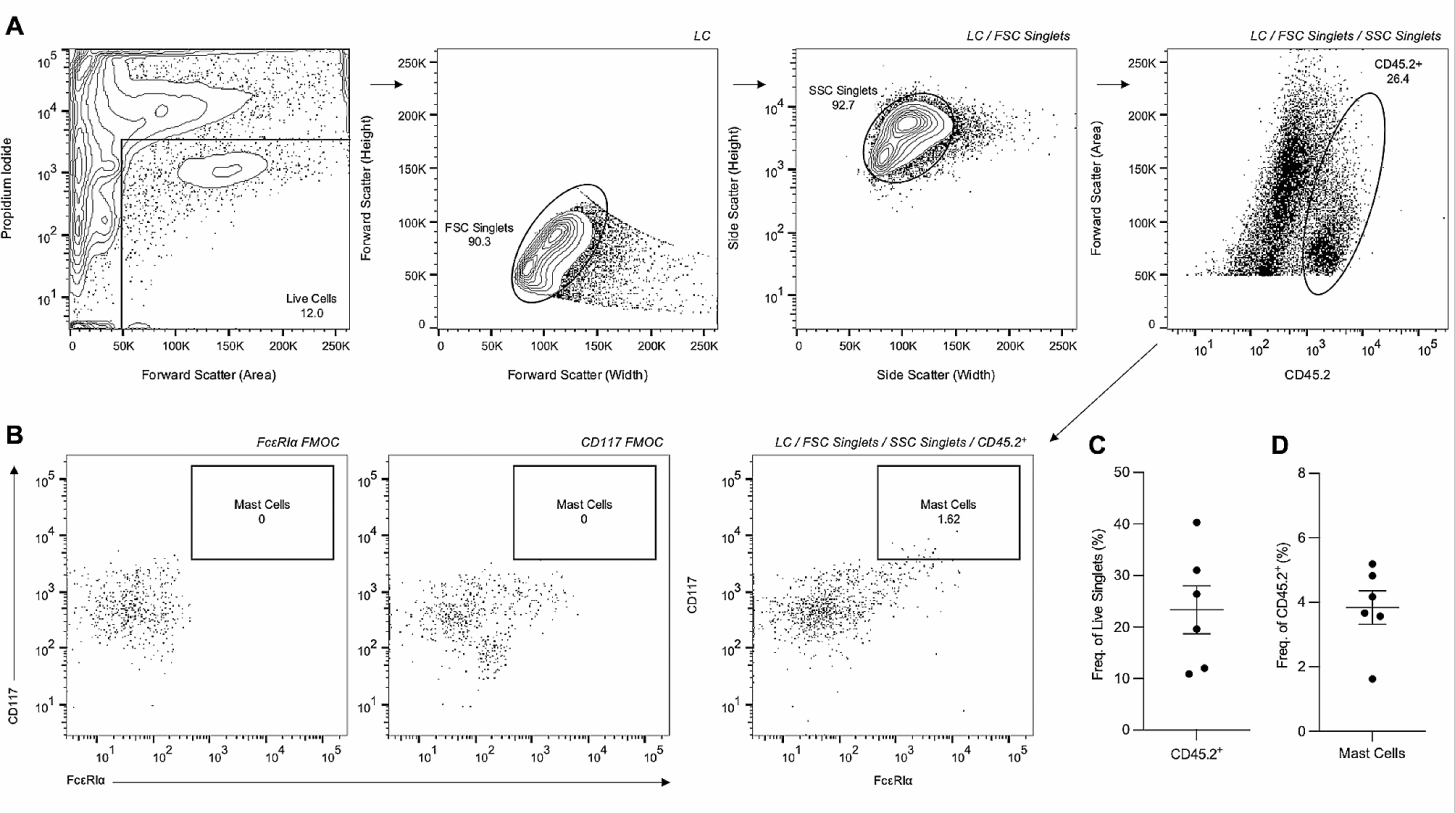
**Identification of urinary bladder mast cells.** (A) Shows identification of live cells (LC) through the exclusion of propidium iodide, followed by forward scatter (FSC) and side scatter (SSC) doublet discrimination and the identification of CD45.2^+^ cells. (B) Shows FMOCs for FcεRIα and CD117 antibodies, which were used to assist the identification urinary bladder mast cells. (C) Shows the frequency of LC/FSC Singlet/SSC Singlet CD45.2^+^ cells and (D) shows mast cell frequency within CD45.2^+^ populations

Single-cell bladder suspensions (2.8 × 10^5^±1.0 × 10^4^ (mean±SEM) cells/bladder) were stained with surface markers described previously to identify mast cells in the urinary bladder. Flow cytometry gating was optimised to minimise the inclusion of debris and dead cells for each sample, as well the incorporation of first minus one control (FMOC) antibody mixtures to determine accurate gate placement. Using this optimised gating strategy, we identified 23.4±4.6% of live urinary bladder cells were CD45.2^+^. From the identified CD45.2^+^ populations, we determined that 3.8±0.5% were mast cells (Fig. 1).

### Mast cell distribution in the urinary bladder

**Fig. 2:**
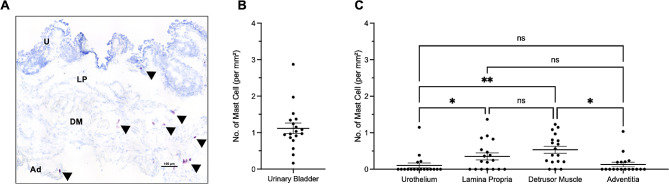
**Mast cell distribution across the urinary bladder wall.** (A) Shows representative image of urinary bladder wall layers, x20 magnification where U = urothelium, LP = lamina propria, DM = detrusor muscle and Ad = adventitia. Mast cells are indicated by black arrowheads. Scale bar represents 100 μm. (B) Shows number of mast cells per mm^2^ of urinary bladder tissue and (C) shows number of mast cells per mm^2^ tissue across the discrete layers of the urinary bladder wall. Each dot represents one section of urinary bladder tissue

Toluidine blue was used to determine mast cell size and distribution throughout the urinary bladder. Across six urinary bladder samples, we determined that mast cells composed 1.1±0.1 (mean±SEM) cells per mm^2^ of bladder tissue. Mast cells were least prevalent in the urothelium (0.1±0.3 cells per mm^2^) and the adventitia (0.13±0.28), followed by the lamina propria (0.3±0.4) and the highest density of mast cells being recorded in the detrusor muscle (0.5±0.4). A repeated measures one-way ANOVA revealed statistical significance in the density of mast cells between the urothelium and lamina propria (*p* ≤ 0.05), the urothelium and detrusor muscle (*p* ≤ 0.005), and the detrusor muscle and adventitia (*p* ≤ 0.05), shown in Fig. 2.

**Fig. 3:**
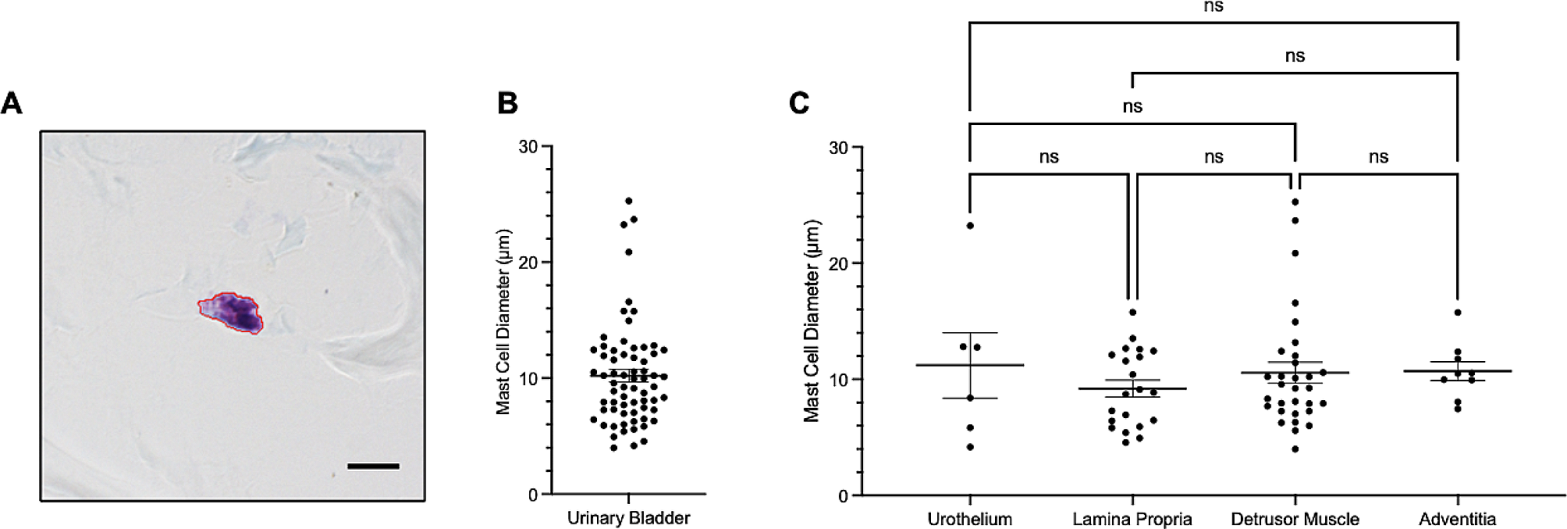
**Mast cell size in the urinary bladder.** (A) Illustrates measured area of mast cell located in the detrusor muscle, x60 magnification. Scale bar represents 10 μm. (B) Shows mast cell diameter and (C) illustrates mast cell size across the layers of the urinary bladder wall. Each dot represents one mast cell

The diameter of mast cells was also measured across the discrete layers of the urinary bladder wall, shown in Fig. 3. On average, the diameter of mast cells was approximately 10.2±0.5 μm, with no statistical difference between the layers of the urinary bladder wall (Fig. 3).

**Fig. 4:**
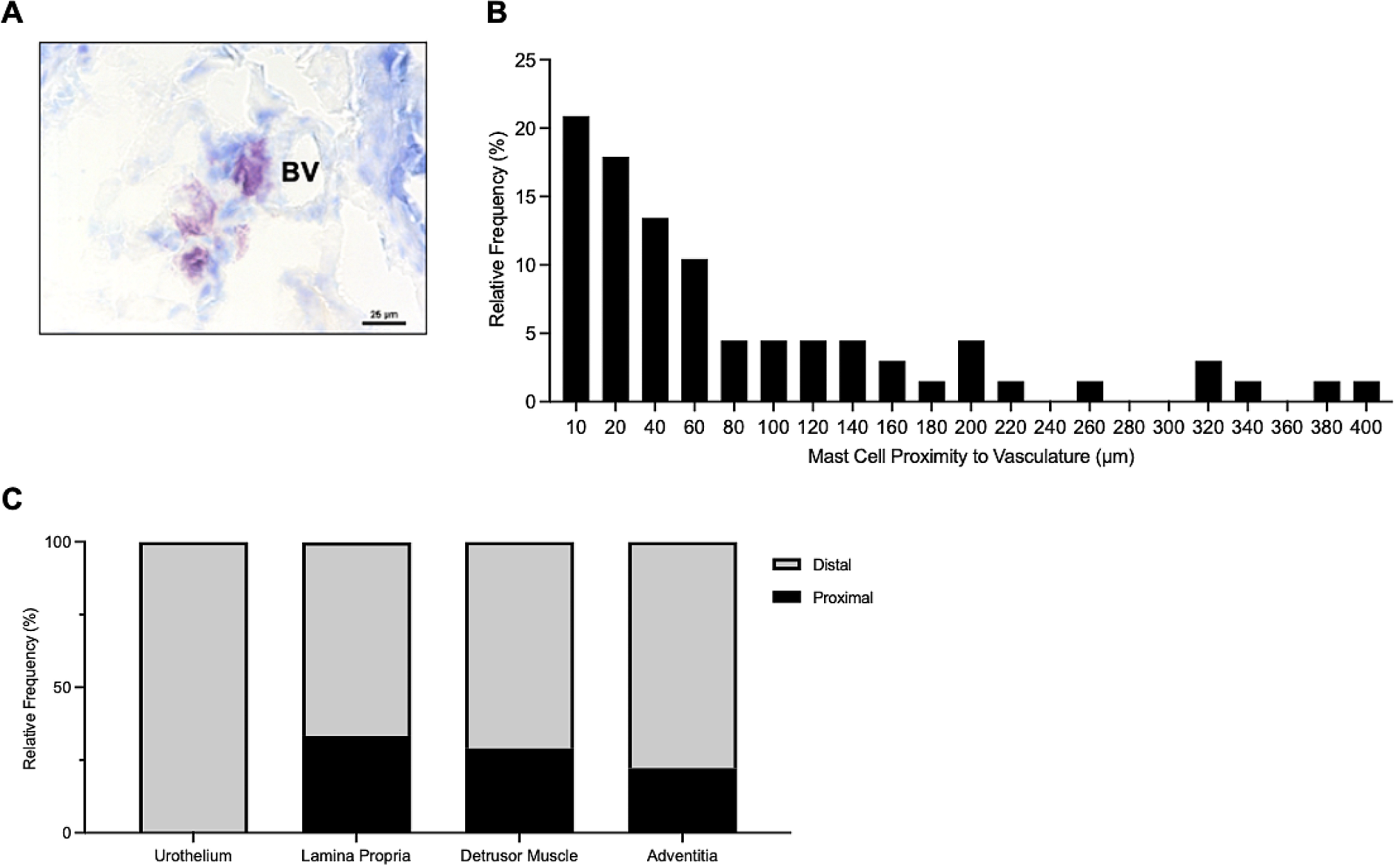
**Mast cells proximal to vasculature in the urinary bladder wall.** (A) shows mast cell proximal to blood vessel (BV) on x40 magnification. Scale bar represents 25 μm. (B) Represents relative frequency of mast cell proximity to vasculature. (C) Displays mast cell proximity to vasculature per urinary bladder layer

The proximity of mast cells to vasculature was also assessed, with 8.9% of total mast cells situated close to blood vessels in the lamina propria, 11.9% in the detrusor layer, and 2.9% in the adventitia (Fig. 4). Mast cells in the urothelium were all considered distal to vasculature. 20.9% of the total mast cells were observed near vasculature across all layers of the urinary bladder wall.

## Discussion

In the urinary bladder, mast cells have been implicated in the pathogenesis of various disorders, such as overactive bladder [9, 10], bladder outlet obstruction [11, 12], urinary tract infections [13], bladder carcinoma [14] and various forms of cystitis [15–18]. Functionally, mast cells are known for their ability to release a wide range of mediators upon activation, which include: histamine, prostaglandin, proteases (tryptase and chymase), cytokines and chemokines such as IL-6, IL-13, chemokine (C-C motif) ligand (CCL)-2, CCL-3, and tumour necrosis factor-α [2, 27–29]. Histamine is one of the best-known mast cell degranulates, and is known to regulate vasodilation and angiogenesis, and is also reported to contribute to the production of prostaglandins and pro-inflammatory cytokines [2]. A recent study by Stromberga, Chess-Williams [30] suggested that histamine can influence contractile activity of the urothelium, lamina propria and detrusor muscle layers through H1 and H2 histamine receptors, with H1 resulting in contraction throughout all discrete layers of the urinary bladder wall, and H2 inhibiting H1-mediated contractions. This data suggests that mast cell-derived histamine may contribute to contractile activity in the urinary bladder, and raises the possibility that increases in mast cell-derived histamine may contribute to contractile dysfunction, seen in overactive bladder [31]. Other degranulates, such as proteases, cytokines and chemokines can: contribute to tissue remodelling [28] and the recruitment of other leukocytes, induce inflammation and fibrosis, and regulate the innate and adaptive immune responses [2, 28, 29]. It is possible that the dysfunction of this multifunctional leukocyte may contribute to the development of disease, evidenced by their implication in a variety of urinary bladder pathologies [19, 22, 32–34]. Despite this, information relating to the distribution of mast cells in the mouse urinary bladder, a common model of urinary bladder diseases, is poorly defined. As such, this study focused on assessing the typical prevalence and distribution of urinary bladder mast cells.

In the present study, we quantified mast cells using flow cytometry based on the expression of their characteristic markers CD45, CD117, and FcɛRIα. We determined that mast cells constituted less than 4% of all leukocytes in the urinary bladder, consistent with previous literature [8]. Our findings provide a baseline understanding of mast cell prevalence in the urinary bladder of mice, which is essential for future studies investigating their involvement in various urinary bladder diseases. This finding also suggests that mast cells compose a relatively rare population of leukocytes in the urinary bladder, which may reflect their functional significance in the healthy urinary bladder.

We also assessed the distribution of mast cells throughout the four layers of the urinary bladder wall, finding that mast cells were more prominent in the lamina propria and detrusor muscle layers. Comparatively, mast cells were less prominent in the urothelium and the adventitia of the urinary bladder. A study conducted by Liu, Shie [10] suggested that mast cells were low in quantity in the urothelium and the lamina propria in humans. However, evidence presented in this study suggests that the urothelium and adventitia exhibit the lowest density of mast cells per mm^2^. Specifically, the lamina propria had a significantly greater density of mast cells (*p* ≤ 0.05) compared to the urothelium in mice. The detrusor muscle also exhibited greater mast cell density per mm^2^ contrast to the adventitia, suggesting that the lamina propria and detrusor muscle layer of the mouse urinary bladder have higher numbers of mast cells. The observation that 20.9% of mast cells were located near vasculature is an interesting finding that may have important implications for understanding mast cell function in the urinary bladder. Physiologically, mast cells are known to regulate blood vessels by releasing vasoactive substances, such as histamine (20, 35). This finding could further indicate mast cell functions, namely extravasation, through its proximity to vasculature; however, further research is needed to validate this conclusion.

Finally, this study also determined that mast cell size did not vary between urinary bladder layers. Previously, mast cell size has been described to indicate cellular heterogeneity and functional activity [36, 37], with Burwen and Satir [36] finding that mast cell radius decreases by ∼ 5% upon stimulation. The aforementioned study suggests that activation of mast cells alters their size, however more work is needed to understand the importance of mast cell morphology in cell heterogeneity and function.

This study has several limitations. Firstly, age and sex are recognised contributors to urinary bladder diseases such as overactive bladder [38] and interstitial cystitis [39], respectively. It is recommended that future studies investigate both age- and sex-related differences in mast cell prevalence and distribution to better understand their physiology and contributions to urinary bladder pathologies. Secondly, the fixative used in this study has been reported to influence mast cell counts [19]. This study suggests that formalin fixation, in combination with the toluidine blue stain, results in poorly stained mast cells which may result in inconsistencies in the number of reported cells. Despite this, formalin is still used in combination with toluidine blue to assess mast cell distribution, and has been used as recently as 2020 [40–42]. Future studies may wish to investigate the mechanism underpinning this phenomenon, and may recommend an alternative fixative for use with toluidine blue. Finally, the method used to investigate mast cell proximity to vasculature requires further validation. For example, future studies may wish to utilise immunohistochemistry to assess the proximity of mast cells (identified by FceRIa and CD117 markers) to an endothelial marker, such as CD31 [43], to affirm the findings of this study.

In summary, our findings demonstrate that mast cells compose less than 4% of all leukocytes in the urinary bladder and are more prominent in the lamina propria and detrusor muscle layers. Additionally, this study determined that mast cell size is consistent throughout all layers of the urinary bladder wall, and that approximately one-fifth of mast cells are proximal to vasculature in the urinary bladder. This finding, in particular, suggests that mast cell proximity to vasculature may be an important factor in their function and subsequent contribution to bladder pathologies. Further studies are needed to elucidate the potential therapeutic implications of targeting mast cells for the treatment of bladder disorders, with particular regard to age- and sex-based differences.

## Data Availability

Data will be made available upon request.
